## [Peer Review File · EMBO Reports]

RNase H1 and Sen1 ensure that transient TERRA R-loops promote the repair of short telomeres

Fabio Bento, Matteo Longaretti, Vanessa Borges Pires, Arianna Lockhart, and Brian Luke

Corresponding author(s): Brian Luke (brialuke@uni-mainz.de)

Review Timeline:

Submission Date:	21st Feb 25
Editorial Decision:	10th Mar 25
Revision Received:	31st Mar 25
Editorial Decision:	4th Apr 25
Revision Received:	17th Apr 25
Accepted:	24th Apr 25

Editor: *Esther Schnapp*

Transaction Report:

Dear Brian,

Thank you for the submission of your manuscript to EMBO reports. We have now received the full set of referee reports that is pasted below.

As you will see, all referees acknowledge that the findings are interesting. However, they also have several comments and suggestions that should all be addressed. Please let me know in case you disagree, and we can discuss the exact revision requirements further, also in a video chat, if you like.

I would thus like to invite you to revise your manuscript with the understanding that the referee concerns must be fully addressed and their suggestions taken on board. Please address all referee concerns in a complete point-by-point response. Acceptance of the manuscript will depend on a positive outcome of a second round of review. It is EMBO reports policy to allow a single round of major revision only and acceptance or rejection of the manuscript will therefore depend on the completeness of your responses included in the next, final version of the manuscript.

We realize that it is difficult to revise to a specific deadline. In the interest of protecting the conceptual advance provided by the work, we recommend a revision within 3 months (10th Jun 2025). Please discuss the revision progress ahead of this time with the editor if you require more time to complete the revisions.

- 1) A data availability section providing access to data deposited in public databases is missing. If you have not deposited any data, please add a sentence to the data availability section that explains that.
- 2) Your manuscript contains statistics and error bars based on $n=2$. Please use scatter blots in these cases. No statistics should be calculated if $n=2$.

5) a complete author checklist, which you can download from our author guidelines . Please insert information in the checklist that is also reflected in the manuscript. The completed author checklist will also be part of the RPF.

6) Please note that all corresponding authors are required to supply an ORCID ID for their name upon submission of a revised

manuscript (). Please find instructions on how to link your ORCID ID to your account in our manuscript tracking system in our Author guidelines

- the name of the statistical test used to generate error bars and P values,
- the number (n) of independent experiments (please specify technical or biological replicates) underlying each data point,
- the nature of the bars and error bars (s.d., s.e.m.),
- If the data are obtained from n {less than or equal to} 2, use scatter blots showing the individual data points.

12) All Materials and Methods need to be described in the main text using our 'Structured Methods' format, which is required for all research articles. According to this format, the Methods section includes a Reagents and Tools Table (listing key reagents, experimental models, software and relevant equipment and including their sources and relevant identifiers) followed by a Methods and Protocols section describing the methods using a step-by-step protocol format. The aim is to facilitate adoption of the methodologies across labs. More information on how to adhere to this format as well as a downloadable template (.docx) for the Reagents and Tools Table can be found in our author guidelines:

An example of a Method paper with Structured Methods can be found here: <https://www.embopress.org/doi/full/10.1038/s44320-024-00037-6#sec-4>

As part of the EMBO publication's Transparent Editorial Process, EMBO reports publishes online a Review Process File (RPF) to accompany accepted manuscripts. This File will be published in conjunction with your paper and will include the referee

reports, your point-by-point response and all pertinent correspondence relating to the manuscript.

I look forward to seeing a revised form of your manuscript when it is ready.

Best,
Esther

Referee #1:

The Luke's laboratory previously showed that RNA-DNA hybrids are present at dysfunctional short telomeres, prevent resection and promote repair by HDR (Pires, 2023). This suggests that TERRA RNA-DNA hybrids require positive and negative regulation to drive repair and are subjected to removal to allow efficient resection and HDR. Therefore, Bento and coworkers further assessed the role of RNase H1 and RNase H2 in telomere maintenance in absence of telomerase.

In this work, they show that RNase H2 binds to normal length telomeres but progressively loses association as telomeres shorten while RNase H1 is recruited when telomeres become critically short, and this occurs as cells enter the S phase. The recruitment of RNase H1 to short telomeres promotes the loading of Rad51, likely by removing the RNA-DNA.

This is an interesting study in line with the previous discoveries of the lab. They shed light on the interplay between RNA-DNA hybrid resolvases at short telomeres, suggesting that RNase H2 is constitutively bound to telomeres and that RNase H1 recruited at short telomere in S phase. Sen1 data are less documented than RNases data.

Comments :

-The constitutive binding of RNase H2 (vs RNase H1) at telomeres is intriguing. Does binding of RNase H2 is cell-cycle regulated like RNaseH1? ChIP of RNase H2 in synchronized cells should be performed.
The authors claim that RNase H2 binding is lost progressively during replicative senescence indicating that telomere length is correlated to the level of RNase H2.
ChIP of RNase H2 in a mutant that have long telomeres could be performed to address this point.

-Recruitment of RNase H1 seems to occur mainly in S phase, likely in response to replication stress at short telomeres. It is shown also that absence or overexpression of RNase H1 accelerate senescence. Absence of RNase H1 also abolishes the binding of Rad51 in est2- cells. Does overexpression of RNaseH1 increase Rad51 binding in est2- cells? How do the authors correlate Rad51 binding with accelerated senescence in absence of RNase H1 and when it is overexpressed?

-The authors claim that "TERRA promotes HDR at short telomeres to prevent early onset of senescence" (p9, second paragraph). What lacks clarity to me is the role of Rad51 during senescence which is depicted in figure 4B. Does Rad51 promotes protection of shortest telomeres or does Rad51 promotes HDR for survivors appearance? Is the model in line with Teixeira lab data that show that Rad51 is crucial to maintain viability in telomerase negative cells? (Xu et al., 2015). Do RNases absence or overexpression have an impact on survivor type?
These points should be clarified.

-The authors investigated Rad51 binding but did not mention RPA recruitment. How is binding of Rad51 correlated to RPA recruitment? If not shown, this could be discuss in the text.

Minor points:

Figure 1A : % of growth reaches 200%. That looks abnormal. Any comments ?

Figure 2D : Is decrease of RNase H2 between PD10 and PD40 statistically significant ? Statistical analysis (left panel) should be presented in comparison with EST2 cells.

Figure 2F: R-ChIP absence of telomerase at PD10 and PD40 would be more informative.

Figure 3: Are ChIP of Sen1 possible in telomerase positive and telomerase negative cells?

Referee #2:

The work by Bento et al describes a study of yeast telomere maintenance examining regulators of delayed replicative senescence. Specifically, TERRA R-loops, which are known to be important for protecting telomeres and initiating telomere recombination are studied. TERRA formation, but also clearance by RNaseH1 and SEN1 is shown to be important for regulating recombination based telomere extension. Thus, the authors expose an elegant regulatory mechanism in which the dynamics of TERRA R-loops provide an important control mechanism, with potential relevance to ALT mechanisms in cancer. Overall the work is interesting and supported by the data. Several suggestions to improve the manuscript are outlined below.

- Figure 1 does not do a very good job presenting any significant differences. This may be a data presentation issue. No measures of statistical significance are shown. Perhaps an informative model cartoon could be juxtaposed, or the absolute value of the nadir in population doublings could be reported and compared? It is interesting that catalytically active and inactive RNH1 have the same effects, I just thought the data could be presented in a more compelling and statistically supported way.

- The senescence curves in Figure 3 have the same problem as Figure 1. The PD Nadir shifts, but its not clear how significant this is and the curves overlap to obscure easy comparison.

-I liked the separation of function allele experiments with SEN1 in Figure 3. Could the authors do the spot dilution growth assays with *sen1-3* also? It would seem fruitful for completeness to observe the suppressive effects on senescence in Fig 3B of *sen1-3* relative to *sen1-1* borne out in the growth assays.

-The Rad51 chip and suppression by RNH1 deletion in Figure 4 is very clear. The discussion on Exo1 mediated resection is quite emphatic but not supported by experiments in this work. Can the authors point to literature, or experiments that I am missing. Could BLM-DNA2, or other nucleases, be involved in initiating HR at these RNH1 processed and shortened telomeres? Perhaps the literature is clear enough, but I would like to at least see some discussion of the other players. Perhaps even better would be redoing the Rad51 ChIP from Figure 4A in an *est2exo1* double mutant to show loss of Rad51. Apologies if this has been shown in previous work that I missed.

Referee #3:

The Luke lab has previously shown that RNase H2 association to telomeres is impaired as telomeres shorten. As a result, TERRA RNA-DNA hybrids accumulate, triggering homology-direct repair (HDR) that delays replicative senescence in the absence of telomerase. In this manuscript, the authors show that RNase H1, in contrast to RNase H2, is recruited to short telomeres to remove RNA-DNA hybrids, which is necessary to promote HDR, presumably because the RNA-DNA hybrids prevent the 5' end resection needed to initiate HDR, as has been demonstrated at double-strand break ends. Overall, this is an important addition to the current knowledge about the role of RNase H1 and H2 on TERRA RNA-DNA hybrids during replicative senescence. Nevertheless, I have a few concerns about the statistical significance of some of the experiments. In particular, I believe that the role of Sen1 during replicative senescence is quite minor (if any at all) compared to RNase H1 and H2.

Specific comments:

The "actin" locus is used a control for ChIP experiments. I assume this is referring to the ACT1 gene? If so, it would be more accurate to say ACT1 instead of actin. Furthermore, why is there less RNase H1 association at this locus in the *est2* PD40 sample (Fig. EV1B)?

In Fig. EV1C, why is senescence delayed in the RNH1-6xHA POL2-9xmyc *est2* strain? For the corresponding ChIP experiments shown in Fig. EV1E, why is statistical significance only indicated when comparing the alpha factor and 30' time points of the RNase H1 ChIP to telomere 15L experiment? Similarly, for Fig. EV1F, why is statistical significance only indicated when comparing the 30' and 45' time points for the Pol2 ChIP to telomere 1L experiment? If none of the other comparisons are statistically significant, the authors should indicate this and modify the text appropriately.

In Fig. 2B (telomere 1L panel), is the difference between "EST2" and "est2 PD40" statistically significant (as it is for the telomere 15L experiment)?

It is stated on page 7 that "Sen1 levels increased, albeit more subtly, at PD40 following telomerase deletion." This is not evident looking at Fig. 2E and no statistical significance is indicated. In addition, it is difficult to conclude that *est2 sen1-1* exhibits accelerated senescence compared to *est2* because the *sen1-1* single mutant is sick at 27C. Taken together, the role of Sen1 on replicative senescence seems rather minor.

In Fig. EV2A, why is there no acceleration of senescence in the *rnh1 est2* strain-as there is in Fig. 3A and as stated in the text?

Referee #1:

Comments :

-The constitutive binding of RNase H2 (vs RNase H1) at telomeres is intriguing. Does binding of RNase H2 is cell-cycle regulated like RNaseH1? ChIP of RNase H2 in synchronized cells should be performed.

We have previously published this in 2017 (Graf et al, Cell, Figure 4B) and shown that H2 binding to telomeres increases throughout the S phase and peaks in late S. This is consistent with TERRA forming hybrids in early S and then the R-loops being removed before the replication machinery passes.

The authors claim that RNase H2 binding is lost progressively during replicative senescence indicating that telomere length is correlated to the level of RNase H2. ChIP of RNase H2 in a mutant that have long telomeres could be performed to address this point.

In figure 4c of Graf et al, we demonstrate that in rif1 mutants where telomeres are extremely long, there is no change in the amount of RNase H2 at telomeres. In rif2 mutants however, there is no binding of RNase H2 to telomeres, despite having long telomeres, consistent with Rif2 interacting with, and binding to, RNase H2.

-Recruitment of RNase H1 seems to occur mainly in S phase, likely in response to replication stress at short telomeres. It is shown also that absence or overexpression of RNase H1 accelerate senescence. Absence of RNase H1 also abolishes the binding of Rad51 in est2- cells. Does overexpression of RNaseH1 increase Rad51 binding in est2- cells? How do the authors correlate Rad51 binding with accelerated senescence in absence of RNase H1 and when it is overexpressed?

The overexpression of RNase H1 reduces Rad51 at short telomeres. This was published in Graf et al, 2017 Cell Figure 2i. Furthermore we showed in Balk et al 2013 that the overexpression of RNase H1 is epistatic with the loss of the HDR machinery. This is consistent with what we are now showing, in that both the appearance and the removal of hybrids are critical for HDR at telomeres. It is known that the loss of RAD51 leads to an increased rate of replicative senescence, because when critically short telomeres arise, they must be repaired (Jolivet et al, Sci. Rep, 2019).

-The authors claim that "TERRA promotes HDR at short telomeres to prevent early onset of senescence" (p9, second paragraph). What lacks clarity to me is the role of Rad51 during senescence which is depicted in figure 4B. Does Rad51 promotes protection of shortest telomeres or does Rad51 promotes HDR for survivors appearance? Is the model in line with Teixeira lab data that show that Rad51 is crucial to maintain viability in telomerase negative cells? (Xu et al., 2015). Do RNases absence or overexpression have an impact on survivor type?

These points should be clarified.

Please see our answer to the point above. Everything depicted is absolutely in line with the Teixeira data in that Rad51 is important to repair short telomeres and prevent early onset replicative senescence. Rad51 is very important for the creation of Type I survivors, however we have not looked into survivor types in this manuscript, but have rather focused on the effects during senescence. We feel that looking at survivors requires an independent study and is beyond the scope of this current manuscript.

-The authors investigated Rad51 binding but did not mention RPA recruitment. How is binding of Rad51 correlated to RPA recruitment? If not shown, this could be discuss in the text.

Thank-you. We agree that this is important to consider and since we speculate that TERRA R-loops are inducing replication stress, it is likely that RPA accumulates. We have included this in the text in the discussion regarding Figure 4. We also comment that RPA has been demonstrated to interact with, and modulate, RNase H1 activity in vivo and in vitro.

Minor points:

Figure 1A : % of growth reaches 200%. That looks abnormal. Any comments ?

We frequently observe this when starting cultures from freshly dissected spores onto selective synthetic media. In the first few days it takes time for cells to adapt from the solid to the liquid media.

Figure 2D : Is decrease of RNase H2 between PD10 and PD40 statistically significant ? Statis tical analysis (left panel) should be presented in comparison with EST2 cells.

It is not, all significant differences are indicated.

Figure 2F: R-ChIP absence of telomerase at PD10 and PD40 would be more informative.

*Performing such experiments in senescent cells has layers of complexity that make it impossible to interpret the results. Indeed, *rnh1*, *rnh201* and *sen1-1* mutants all senesce at very different rates, therefor we cannot directly assess whether R-loops are due to the loss of the gene or the different stage of senescence, hence we have decided to perform the R-ChIP in telomerase positive cells.*

Figure 3: Are ChIP of Sen1 possible in telomerase positive and telomerase negative cells?

Please see Figure 2E, we have performed the Sen1 ChIP in EST2 and est2 cells.

Referee #2:

- Figure 1 does not do a very good job presenting any significant differences. This may be a data presentation issue. No measures of statistical significance

are shown. Perhaps an informative model cartoon could be juxtaposed, or the absolute value of the nadir in population doublings could be reported and compared? It is interesting that catalytically active and inactive RNH1 have the same effects, I just thought the data could be presented in a more compelling and statistically supported way.

Thanks for pointing this out. We have included a cartoon as you suggested to clarify what we are seeing on the senescence curves (see new Figure 1A). Furthermore we have performed statistical test between the values at the NADIR, which we have now included in tables beside each curve. I think this will make the data much more accessible for the reader.

- The senescence curves in Figure 3 have the same problem as Figure 1. The PD Nadir shifts, but its not clear how significant this is and the curves overlap to obscure easy comparison.

-see answer to comment above

-I liked the separation of function allele experiments with SEN1 in Figure 3. Could the authors do the spot dilution growth assays with *sen1-3* also? It would seem fruitful for completeness to observe the suppressive effects on senescence in Fig 3B of *sen1-3* relative to *sen1-1* borne out in the growth assays.

-We have performed this analysis as suggested, and included it as Figure 3D. The results mirrors what we reported in Figure 3B and is consistent with the liquid assay.

-The Rad51 chip and suppression by RNH1 deletion in Figure 4 is very clear. The discussion on Exo1 mediated resection is quite emphatic but not supported by experiments in this work. Can the authors point to literature, or experiments that I am missing. Could BLM-DNA2, or other nucleases, be involved in initiating HR at these RNH1 processed and shortened telomeres? Perhaps the literature is clear enough, but I would like to at least see some discussion of the other players. Perhaps even better would be redoing the Rad51 ChIP from Figure 4A in an *est2exo1* double mutant to show loss of Rad51. Apologies if this has been shown in previous work that I missed.

- you are correct, we were much too exclusive in terms of only referring to Exo1. We have expanded the discussion here in the description of the Figure 4 data. We largely refer to the Fallet et al, NAR 2014 paper from the Teixeira lab. In this paper it was demonstrated that in addition to Exo1, also Sae2, Mre11 and Sgs1 are involved in processing short telomeres. Indeed, they all contribute to the generation of ssDNA at telomeres which then gets loaded with Rad51 and Rad52. In this paper, they show that an experimentally induced short telomere accumulates ssDNA in an Mre11 and Exo1 dependent manner. Hence will will tone down the emphasis specifically on Exo1 inhibition and rather refer to "resection factors" with reference to this publication. The model will also be adjusted accordingly.

Referee #3:

Specific comments:

The "actin" locus is used a control for ChIP experiments. I assume this is referring to the ACT1 gene? If so, it would be more accurate to say ACT1 instead of actin. Furthermore, why is there less RNase H1 association at this locus in the est2 PD40 sample (Fig. EV1B)?

-Thanks for this comment. Indeed, we have re-labelled this correctly as ACT1. Although it is not statistically different, there is less RNase H1 not only at ACT1 in PD10, but also at telomeres in PD10. We don't have an explanation for this yet, but we are working on it. What I can say is that Rnh1 levels decrease as cells enter senescence, as measured by western blotting (see below figure). Hence the decrease at PD10 may account for the decreased binding. At PD40 we also have decreased H1 levels (below), but now we have local replication stress at telomeres, and this likely accounts for the accumulation and recruitment at telomeres despite the decreased levels. This is work in progress and will be published elsewhere, where we describe how the DNA damage checkpoint affects RNase H1 levels and recruitment.

Figures for referees not shown.

In Fig. EV1C, why is senescence delayed in the RNH1-6xHA POL2-9xmyc est2 strain?

-we believe that there may be an effects of the epitope tags, likely from the Pol2-9xmyc strain. We know that the Rnh1-HA tag seems to function normally, and the Pol2-9xmyc is viable, but it may have slight replication alterations that are not detectable on the level of viability. We have now commented on this in the text and acknowledge that the epitope tags may affect senescence rates.

For the corresponding ChIP experiments shown in Fig. EV1E, why is statistical significance only indicated when comparing the alpha factor and 30' time

points of the RNase H1 ChIP to telomere 15L experiment? Similarly, for Fig. EV1F, why is statistical significance only indicated when comparing the 30' and 45' time points for the Pol2 ChIP to telomere 1L experiment? If none of the other comparisons are statistically significant, the authors should indicate this and modify the text appropriately.

-we have gone through all figures and improved how we report the statistical significance as requested by reviewer 2. In short, all significant differences are now indicated along with the corresponding p value. For Figure EV1F, the differences are not statistically different and we have modified the text accordingly to point out that there were culture to culture differences in terms of synchrony. We have corrected a calculation error on the FigEV1F for the alpha-factor sample, however this does not change the outcome of the results.

In Fig. 2B (telomere 1L panel), is the difference between "EST2" and "est2 PD40" statistically significant (as it is for the telomere 15L experiment)?

-it is not, all statistics data is now available on the plotted graphs

It is stated on page 7 that "Sen1 levels increased, albeit more subtly, at PD40 following telomerase deletion." This is not evident looking at Fig. 2E and no statistical significance is indicated.

-agree, that at telomere 1L the increase is not as striking as it is at 15L. Nonetheless it is significantly increased when comparing early to late population doublings at both telomeres that we analyzed.

In addition, it is difficult to conclude that est2 sen1-1 exhibits accelerated senescence compared to est2 because the sen1-1 single mutant is sick at 27C. Taken together, the role of Sen1 on replicative senescence seems rather minor.

-we can only agree that the effects of the sen1-1 mutant are more difficult to interpret due to the sickness at 27°C however the delayed senescence of the est2 rnh201 mutant is clearly reversed by the sen1-1 mutant, as the est2 rnh201 sen1-1 looks identical to the est2 sen1-1. In addition we also see the reversal of delayed senescence in rnh201 est2 mutants with the sen1-3 mutant and this is not temperature dependent. We have now also included a spotting to show this in a complementary manner as was requested by reviewer 2.

In Fig. EV2A, why is there no acceleration of senescence in the rnh1 est2 strain-as there is in Fig. 3A and as stated in the text?

-We agree, that in this particular experiment we did not see a big difference in the rate of senescence. However we have repeated this experiment and can confirm that the rnh1 mutant is fast-senescent (see Figure below from an independent heterozygous diploid). This is a bit of a problem with these liquid growth experiments as we are artificially selecting for the best growers which often prevail and there are culture to culture variations. It is also important to point out that because of this problem we normally do at least 6 biological replicates, but since we were also doing ChIP in the experiment you were referring to, we only did 3 replicates. However, we feel it is important to show this particular curve as it corresponds to

the Rad51 ChIP experiments that were performed. So even though the senescence rates were not strongly affected in this experiment, we can still detect differences in Rad51 levels.

Dear Brian,

Thank you for the submission of your revised manuscript. We have now received the enclosed report from referee 1 who was asked to assess it. This referee only has one more minor comment that I would like you to address before we can proceed with the official acceptance of your manuscript.

A few editorial requests will also need to be addressed:

- The Data Availability section needs to be placed before the Acknowledgments
- There is an author name discrepancy: Vanessa Borges Pires in the ms vs. Vanessa Pires in the online submission system, please correct.
- The author CHECKLIST file is missing the corr. author name, journal name and ms ID#; it appears that there is an error with the Excel file (pop-up window: Some parts of this workbook may have been repaired or discarded.) It may be best to download a new checklist and submit it with your final ms. Also, the entire section on statistics needs to be filled in.
- Tables EV1-EV3 have the following titles in their files: Table S1-S3, this needs to be corrected especially the mismatch between the numbers of the tables (e.g. Table EV2 has Table S3 title, etc.); Table EV2 and EV3 are only called out in the Reagents&Tools table which needs to be removed from the ms; we need the callouts for Table EV2 and EV3 to also be provided in the ms text. The Reagents&Tools table needs to be uploaded as an individual file.
- The manuscript sections should be in the following order: Title page - Abstract & Keywords - Introduction - Results - Discussion - Methods - Data Availability - Acknowledgments - Disclosure Statement & Competing Interests - References - Figure Legends - (Main Tables with legends if applicable) - Expanded View Figure Legends

Figure Legends - Comments

- Please note that the exact p values are not provided in the legend of figure 4A, please provide exact values as reasonable.
- Please note that the legends for figure 2 is not provided in the sequential manner (legend for sub-figure 2D is provided before legend of sub-figure 2B, C). This needs to be rectified.
- Please note that the legends for figure 3 is not provided in the sequential manner (legend for sub-figure 2C is provided before legend of sub-figure 3B). This needs to be rectified.

EMBO press papers are accompanied online by A) a short (1-2 sentences) summary of the findings and their significance, B) 2-3 bullet points highlighting key results and C) a synopsis image that is exactly 550 pixels wide and 200-600 pixels high (the height is variable). The synopsis image should provide a sketch of the major findings, like a graphical abstract. Please note that text needs to be readable at the final size. Please send us this information along with the final manuscript.

Best,
Esther

Referee #1:

The authors addressed the concerns of all referees. I would recommend that certain points addressed in the point-by-point response and referring to previously published results by the Luke's lab and the Teixeira's lab, be added to the text for a better understanding by a wider readership

Referee #1:

Comments :

The authors addressed the concerns of all referees. I would recommend that certain points addressed in the point-by-point response and referring to previously published results by the Luke's lab and the Teixeira's lab, be added to the text for a better understanding by a wider readership

Thanks for taking the time to go through our revisions. With regards to your remaining comment, we have now added more detail regarding the regulation of RNase H2 at telomeres and how it regulates TERRA RNA-DNA hybrids. This can be found in the introduction. In the discussion we have expanded on the known Teixeira data implicating Rad51 and the production of ssDNA at critically short telomeres.

Prof. Brian Luke
Johannes Gutenberg University
Institute of Developmental Biology and Neurobiology
Hanns-Dieter-Hüsch-Weg 15
Mainz 55128
Germany

Dear Brian,

I am very pleased to accept your manuscript for publication in the next available issue of EMBO reports. Thank you for your contribution to our journal.
