# Supplementary material for: RNase H1 and Sen1 ensure that transient TERRA R-loops promote the repair of short telomeres
Source: EMBO Rep. 2025 May 22;26(12):3032–44. doi: 10.1038/s44319-025-00469-7 (PMC12187912; doi:10.1038/s44319-025-00469-7)
Supplement: Supplementary file 1 — Table EV1 [file 44319_2025_469_MOESM1_ESM.pdf]

Table EV1. Yeast Strains used in t

Strain name
yAL761
yAM186
yBB236
yBL385
yBL461
yCW1598
yFB2495
yFB2570
yFB2576
yFB2588
yTW501
yVP127
yVP895
yVP1116
yML1,2, 3
yML4, 5, 6
yML7, 8, 9
yML10, 11, 12
yML13, 14, 15
yML16, 17, 18
yML19, 20, 21
yML22, 23, 24
yML25, 26, 27
yML28, 29, 30
yML31, 32, 33
yML34, 35, 36
yML37, 38, 39
yML40, 41, 42
yML43, 44, 45
yML46, 47, 48

this study

Genotype
MATa his3Δ1; leu2Δ0; ura3Δ0; met15Δ0;rnh1::HYG rnh201::NAT
MATa/MATalpha his3Δ1/his3Δ1 leu2Δ0/leu2Δ0 ura3Δ0/ura3Δ0 met15Δ0/met15Δ0 RNH2/rnh201::hyg RAD52/rad
MATa/MATalpha his3Δ1/his3Δ1 leu2Δ0/leu2Δ0 ura3Δ0/ura3Δ0 MET15/met15Δ0 RNH1/rnh1::kan RNH2/rnh2::nat
MATalpha his3Δ1 leu2Δ0 ura3Δ0 est2::kan
MATalpha his3Δ1 leu2Δ0 ura3Δ0 met15Δ0 est2::NAT
MATa his3Δ1; leu2Δ0; met15Δ0; ura3Δ0; RNH1-6xHA-KAN; sen1-3-9xMyc-His
MATa/MATalpha; his3Δ1/ his3Δ1;leu2Δ0 /leu2Δ0; ura3Δ0/ura3Δ0; RNH201/RNH201-6xmyc::HIS RNH1-6xHA::KA
MATa his3Δ1; leu2Δ0; ura3Δ0; met15Δ0; Pol2-3xHA-HYG Sen1-9xmyc-NAT
MATa his3Δ1; leu2Δ0; ura3Δ0; met15Δ0; Pol2-9xmyc-AID-HYG RNH1-6xHA::KAN
MATalpha his3Δ1; leu2Δ0; ura3Δ0; met15Δ0; sen1-3-myc::HIS est2::KAN
MATa/MATalpha; his3Δ1/his3Δ1; leu2Δ0/leu2Δ0; ura3Δ0/ ura3Δ0; LYS2/lys2Δ0; MET15/met15Δ0; EST2/est2::KAN
MATalpha his3Δ1 leu2Δ0 ura3Δ0 met15Δ0 rnh1::HIS; rnh201::HYG
MATa/MATalpha; his3Δ1/his3Δ1; leu2Δ0/leu2Δ0; ura3Δ0/ura3Δ0; met15Δ0/met15Δ0; EST2/est2::NAT; RNH1/rnh
MATalpha his3Δ1 leu2Δ0 ura3Δ0 met15Δ0 est2::KAN rnh201::HYG
MAT a his3Δ1 leu2Δ0 met15Δ0 ura3Δ0 + pBL 959
MAT a his3Δ1 leu2Δ0 met15Δ0 ura3Δ3 + pBL967
MAT a his3Δ1 leu2Δ0 met15Δ0 ura3Δ0 rnh1::KAN + pBL 959
MAT a his3Δ1 leu2Δ0 met15Δ0 ura3Δ0 rnh1::KAN + pBL967
MAT a his3Δ1 leu2Δ0 met15Δ0 ura3Δ0 rnh201::KAN + pBL 959
MAT a his3Δ1 leu2Δ0 met15Δ0 ura3Δ0 rnh201::KAN + pBL967
MAT a his3Δ1 leu2Δ0 met15Δ0 ura3Δ0 rnh1::KAN rnh201::HYG + pBL 959
MAT a his3Δ1 leu2Δ0 met15Δ0 ura3Δ0 rnh1::KAN rnh201::HYG + pBL967
MAT a his3Δ1 leu2Δ0 met15Δ0 ura3Δ0 GPD-AFB2 (LEU) Sen1::AID-MYC-HIS + pBL 959
MAT a his3Δ1 leu2Δ0 met15Δ0 ura3Δ0 GPD-AFB2 (LEU) Sen1::AID-MYC-HIS + pBL967
MAT a his3Δ1 leu2Δ0 ura3Δ0 rnh1::KAN sen1::AID-MYC-HIS GPD-AFB2 (LEU) + pBL 959
MAT a his3Δ1 leu2Δ0 ura3Δ0 rnh1::KAN sen1::AID-MYC-HIS GPD-AFB2 (LEU) + pBL967
MAT alpha his3Δ1 leu2Δ0 ura3Δ0 rnh201::HYG sen1::AID-MYC-HIS GPD-AFB2 (LEU) + pBL 959
MAT alpha his3Δ1 leu2Δ0 ura3Δ0 rnh201::HYG sen1::AID-MYC-HIS GPD-AFB2 (LEU) + pBL967
MAT a his3Δ1 leu2Δ0 ura3Δ0 rnh1::KAN rnh201::HYG sen1::AID-MYC-HIS GPD-AFB2 (LEU) + pBL 959
MAT a his3Δ1 leu2Δ0 ura3Δ0 rnh1::KAN rnh201::HYG sen1::AID-MYC-HIS GPD-AFB2 (LEU) + pBL967

Source
This study
This study
This study
This study
This study
This study
This study
This study
This study
This study
This study
This study
This study
This study
This study
This study
This study
This study
This study
This study
This study
This study
This study
This study
This study
This study
This study
This study
This study
This study

Yeast strains used in this study
