# Supplementary material for: RNase H1 and Sen1 ensure that transient TERRA R-loops promote the repair of short telomeres
Source: EMBO Rep. 2025 May 22;26(12):3032–44. doi: 10.1038/s44319-025-00469-7 (PMC12187912; doi:10.1038/s44319-025-00469-7)
Supplement: Supplementary file 2 — Table EV2 [file 44319_2025_469_MOESM2_ESM.pdf]

Table EV2: Plasmids used in this study

Plasmid identifier	Plasmid name
pBL837	pRS416 pGal-RNH1(D193N)-HA
pBL906	pRS416 pGal-3HA
pBL907	pRS416 pGPD-3HA
pBL908	pRS416 pGPD-RNH1-3HA
pBL923	pRS416 pGPD-RNH1(D193N)-3HA
pBL930	pRS426 pGPD-3HA
pBL931	pRS426 pGPD-RNH1-3HA
pBL938	pRS426 pGPD-RNH1(D193N)-3HA
pBL959	pRS426-pGAL-3HA
pBL967	pRS426-pGAL-RNH1(D193N)-3HA

Source
Misino et al., 2022
Misino et al., 2022
This study
This study
This study
This study
This study
This study
This study
This study
