# Supplementary material for: RNase H1 and Sen1 ensure that transient TERRA R-loops promote the repair of short telomeres
Source: EMBO Rep. 2025 May 22;26(12):3032–44. doi: 10.1038/s44319-025-00469-7 (PMC12187912; doi:10.1038/s44319-025-00469-7)
Supplement: Supplementary file 3 — Table EV3 [file 44319_2025_469_MOESM3_ESM.pdf]

Table EV3: Oligonucleotides used in this study

Name	Sequence
oBL292	CCCAGGTATTGCCGAAAGAATGC
oBL293	TTTGTTGGAAGGTAGTCAAAGAAGCC
oBL295	CGGTGGGTGAGTGGTAGTAAGTAGA
oBL296	ACCCTGTCCCATTCAACCATAC
oLK57	GGGTAACGAGTGGGGAGGTAA
oLK58	CAACACTACCCTAATCTAACCCTGT

Target gene	Direction	Application
Actin	Forward	qPCR
Actin	Reverse	qPCR
Telomere 1L	Forward	qPCR
Telomere 1L	Reverse	qPCR
Telomere 15L	Forward	qPCR
Telomere 15L	Reverse	qPCR

Source

qPCR oligosnuclotides used in this study
