# Supplementary figures and images for: RNase H1 and Sen1 ensure that transient TERRA R-loops promote the repair of short telomeres
Source: EMBO Rep. 2025 May 22;26(12):3032–44. doi: 10.1038/s44319-025-00469-7 (PMC12187912; doi:10.1038/s44319-025-00469-7)

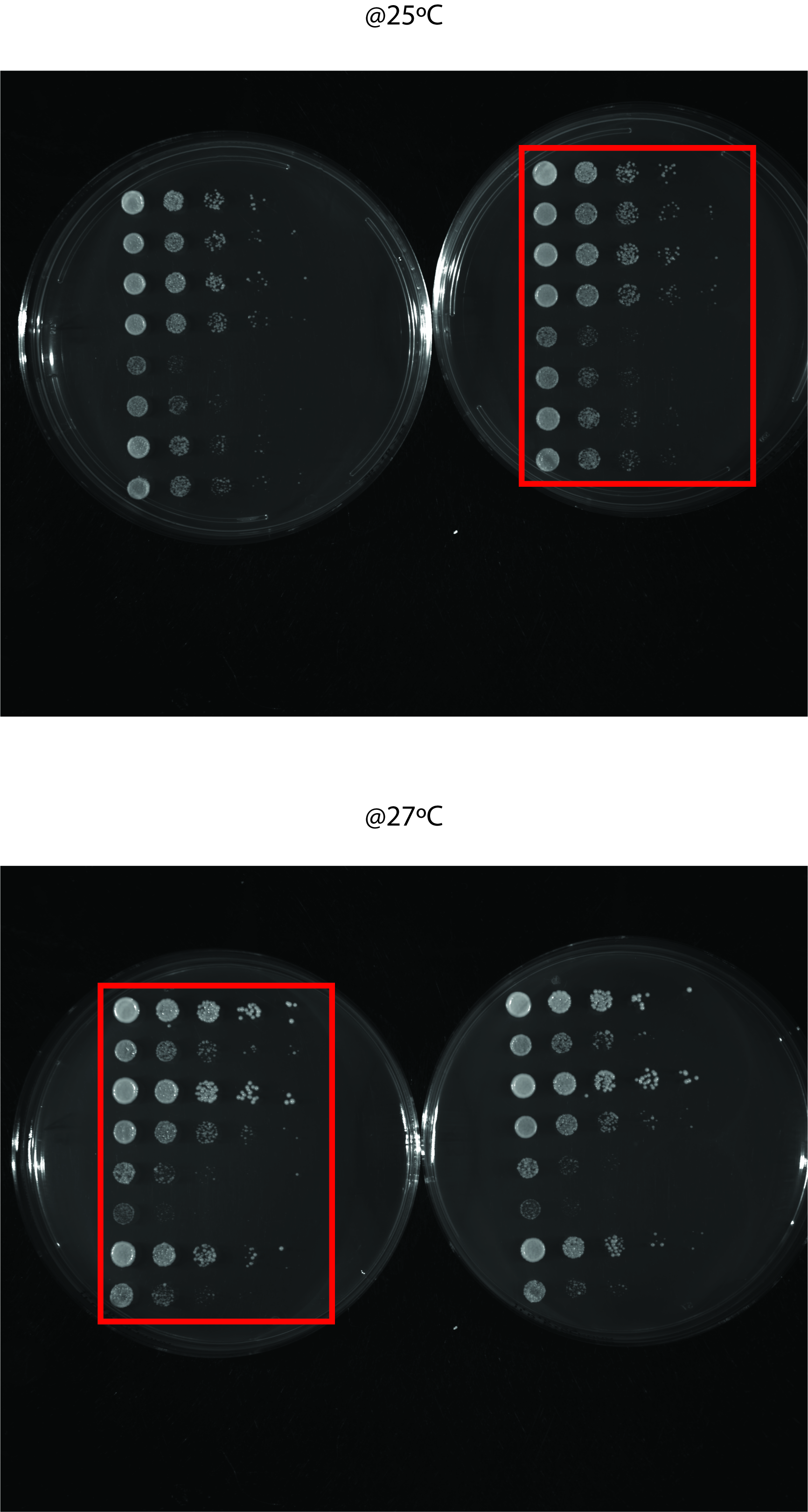

Supplement: Supplementary file 7 — Source data Fig. 3 [file 44319_2025_469_MOESM7_ESM.zip › Figure 3/3B/Passage 4.tif]

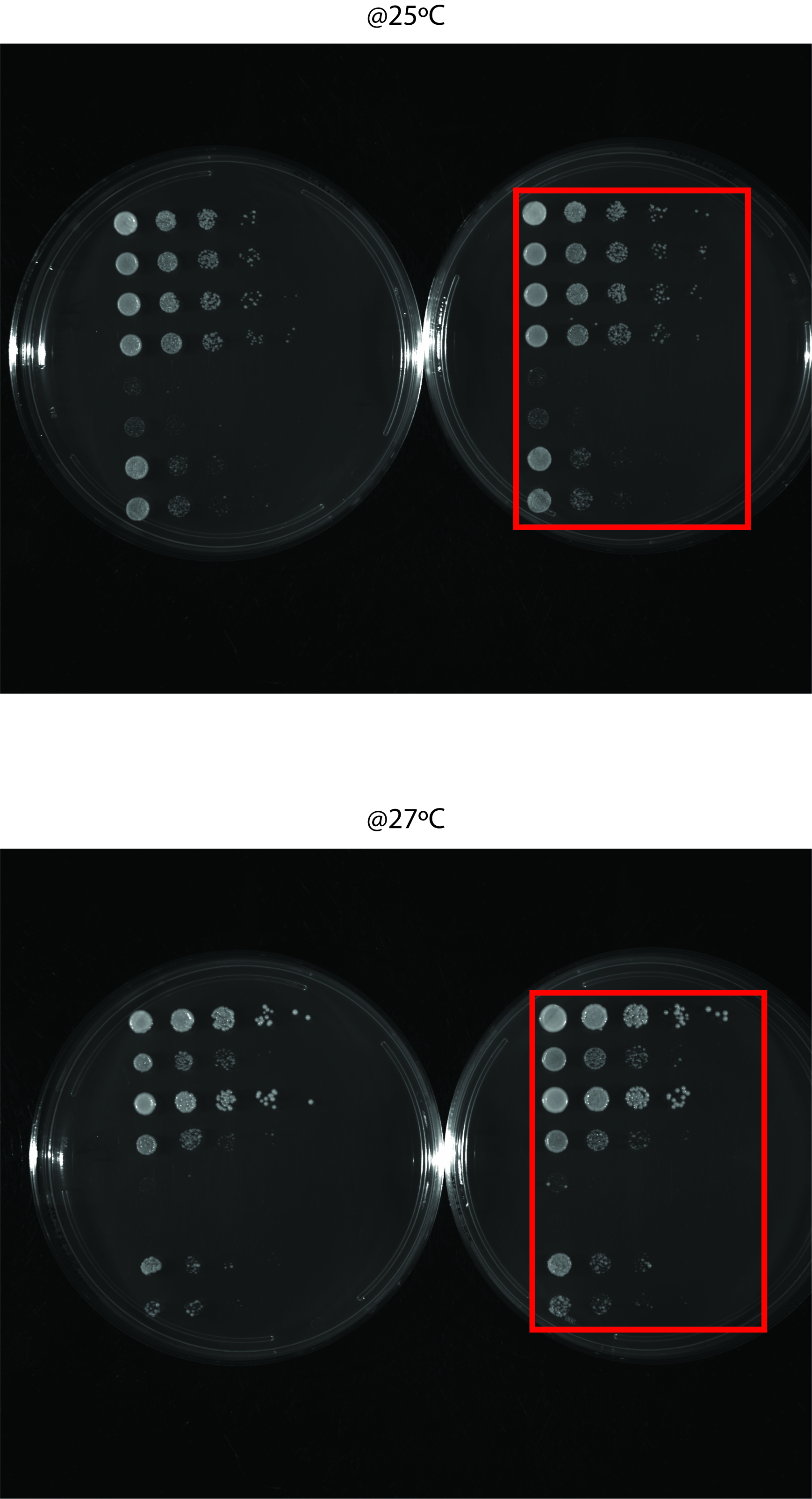

Supplement: Supplementary file 7 — Source data Fig. 3 [file 44319_2025_469_MOESM7_ESM.zip › Figure 3/3B/Passage 5.tif]

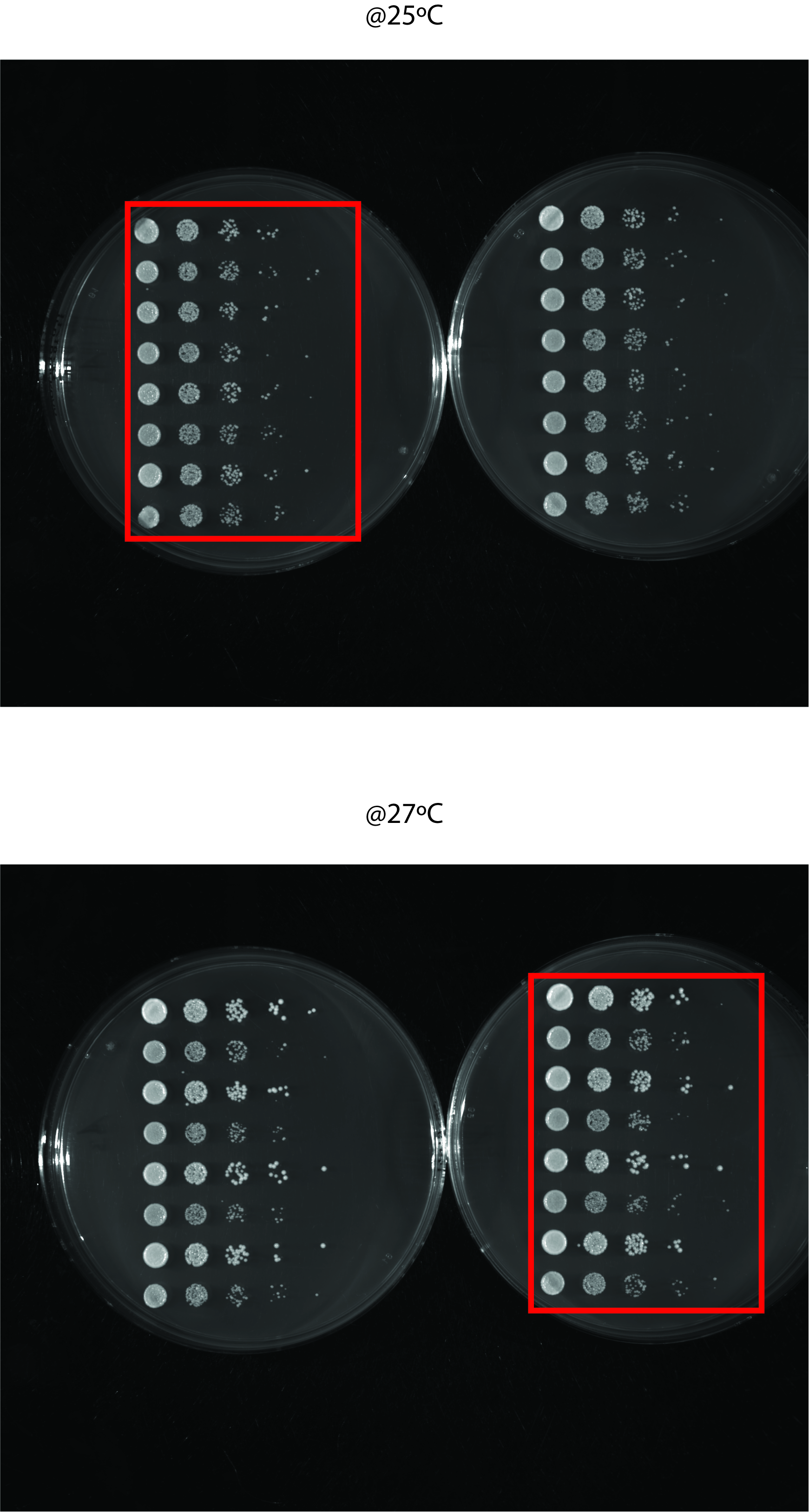

Supplement: Supplementary file 7 — Source data Fig. 3 [file 44319_2025_469_MOESM7_ESM.zip › Figure 3/3B/Passage 1.tif]

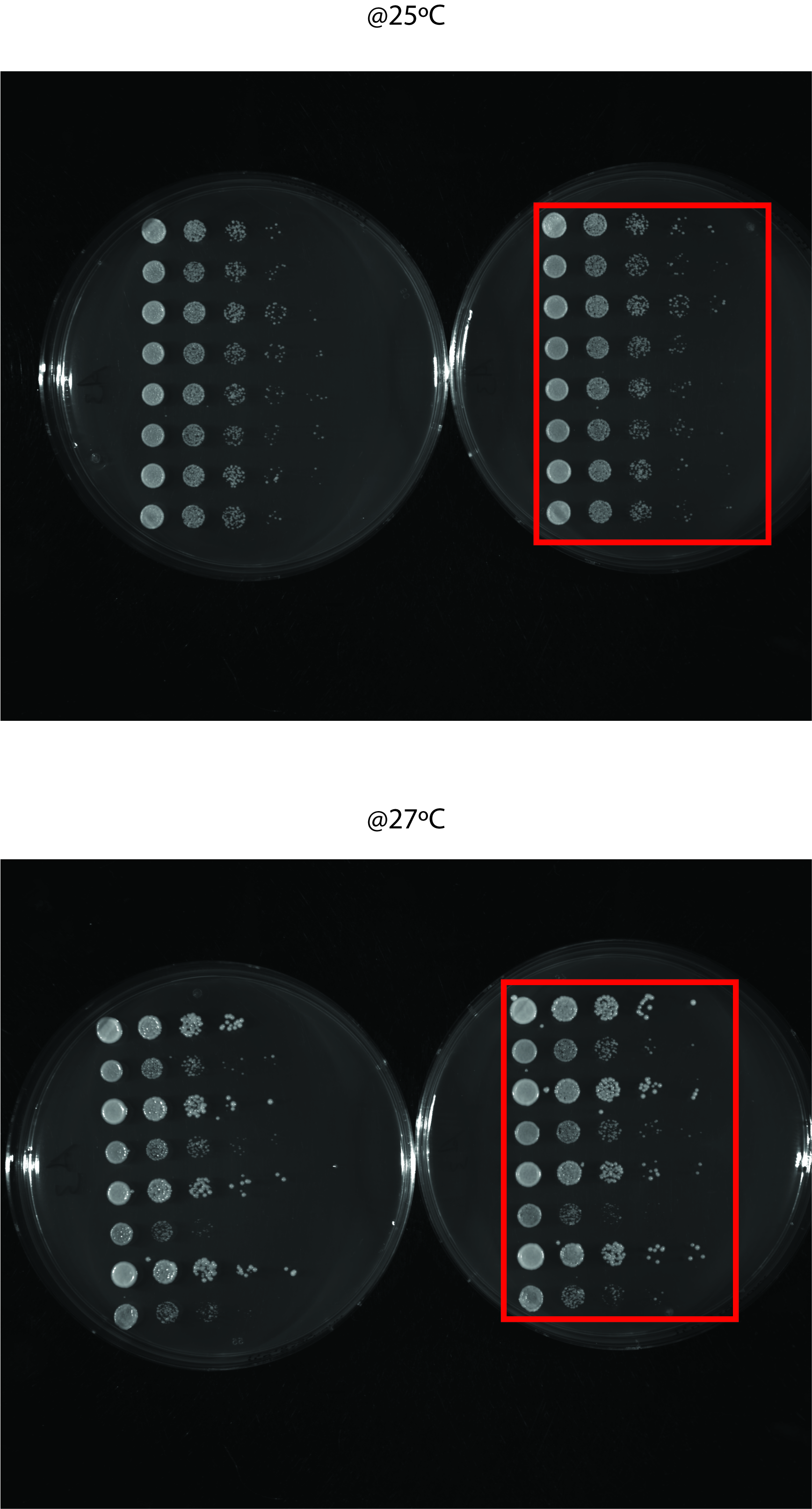

Supplement: Supplementary file 7 — Source data Fig. 3 [file 44319_2025_469_MOESM7_ESM.zip › Figure 3/3B/Passage 2.tif]

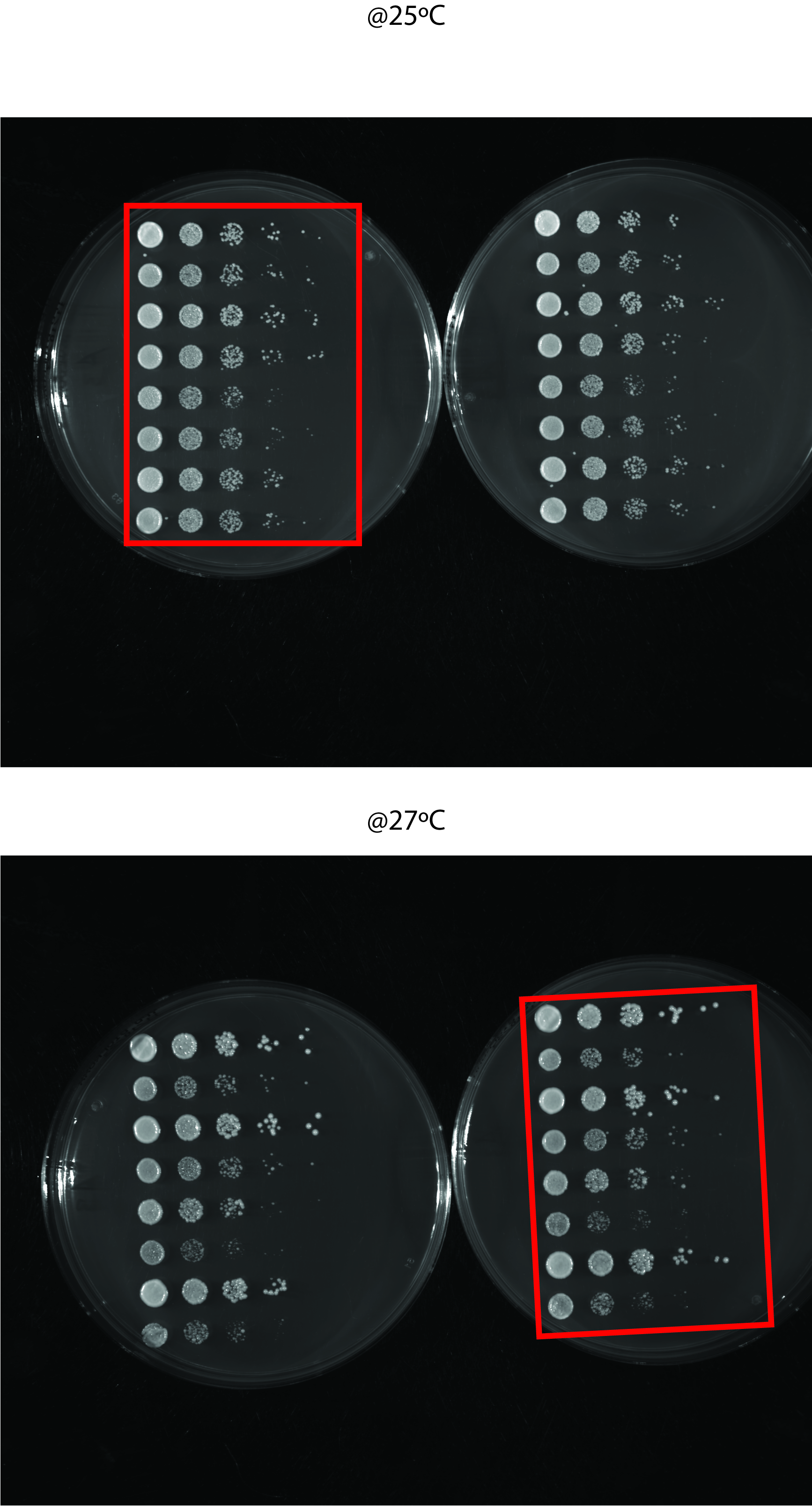

Supplement: Supplementary file 7 — Source data Fig. 3 [file 44319_2025_469_MOESM7_ESM.zip › Figure 3/3B/Passage 3.tif]

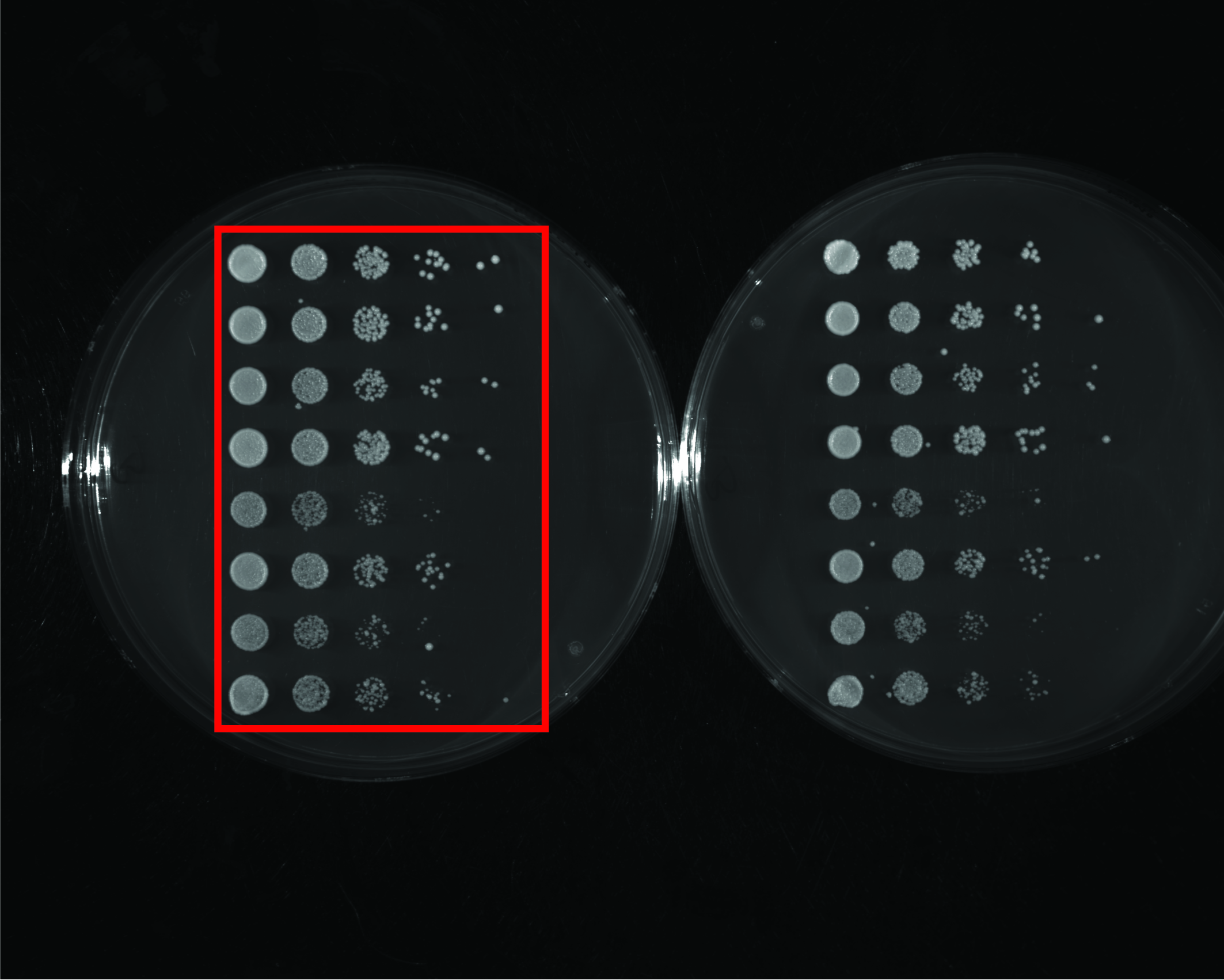

Supplement: Supplementary file 7 — Source data Fig. 3 [file 44319_2025_469_MOESM7_ESM.zip › Figure 3/3D/Passage 4.tif]

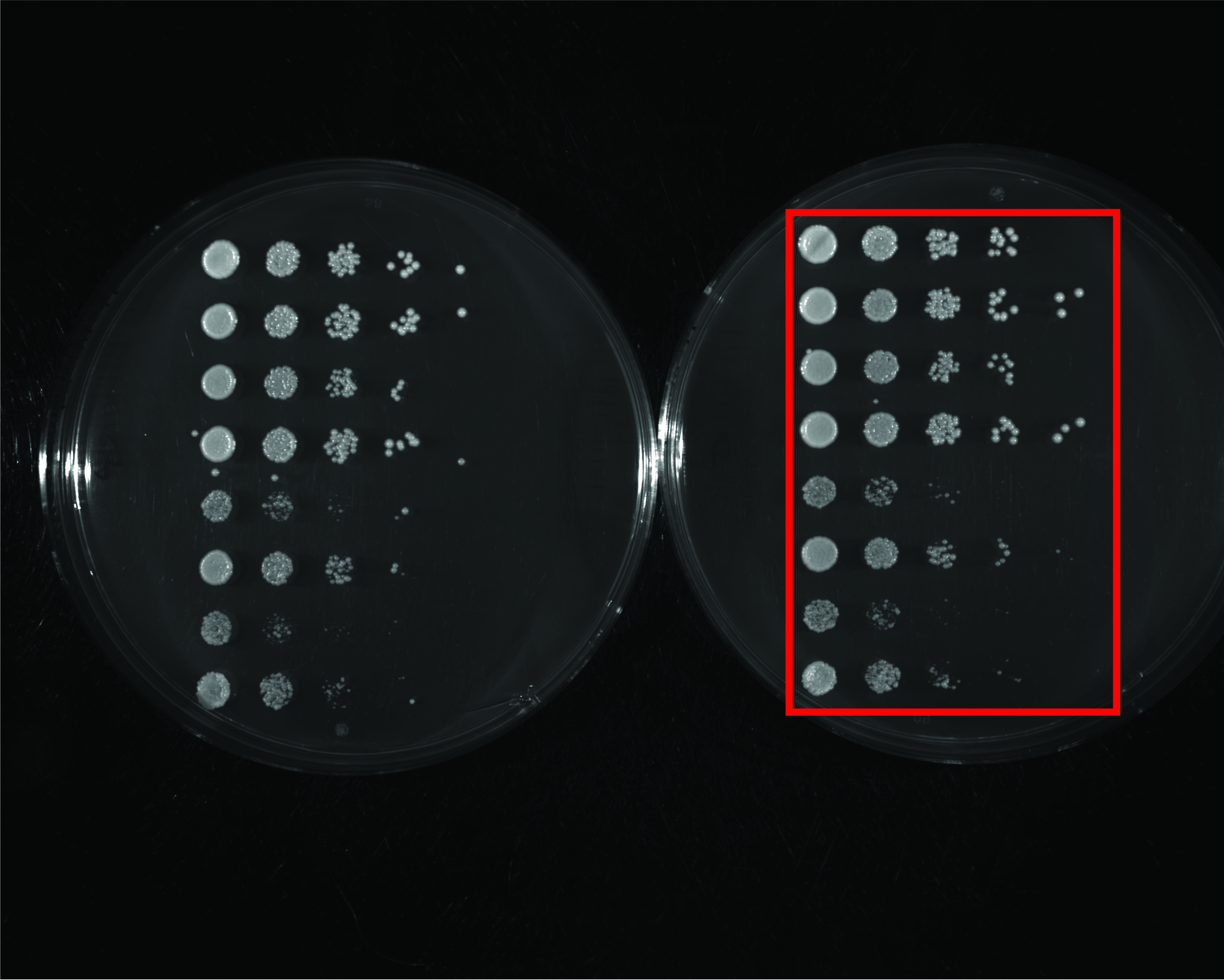

Supplement: Supplementary file 7 — Source data Fig. 3 [file 44319_2025_469_MOESM7_ESM.zip › Figure 3/3D/Passage 5.tif]

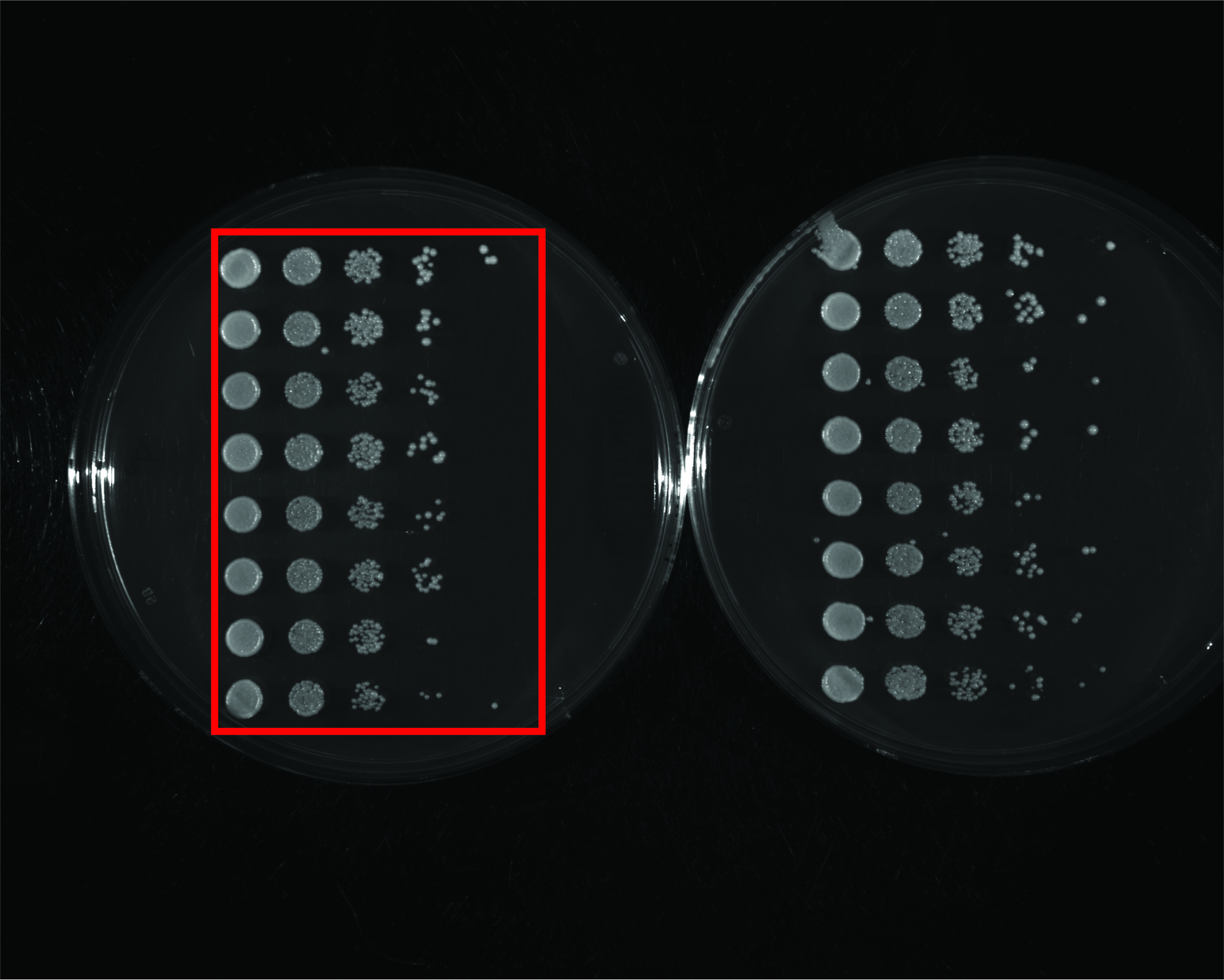

Supplement: Supplementary file 7 — Source data Fig. 3 [file 44319_2025_469_MOESM7_ESM.zip › Figure 3/3D/Passage 1.tif]

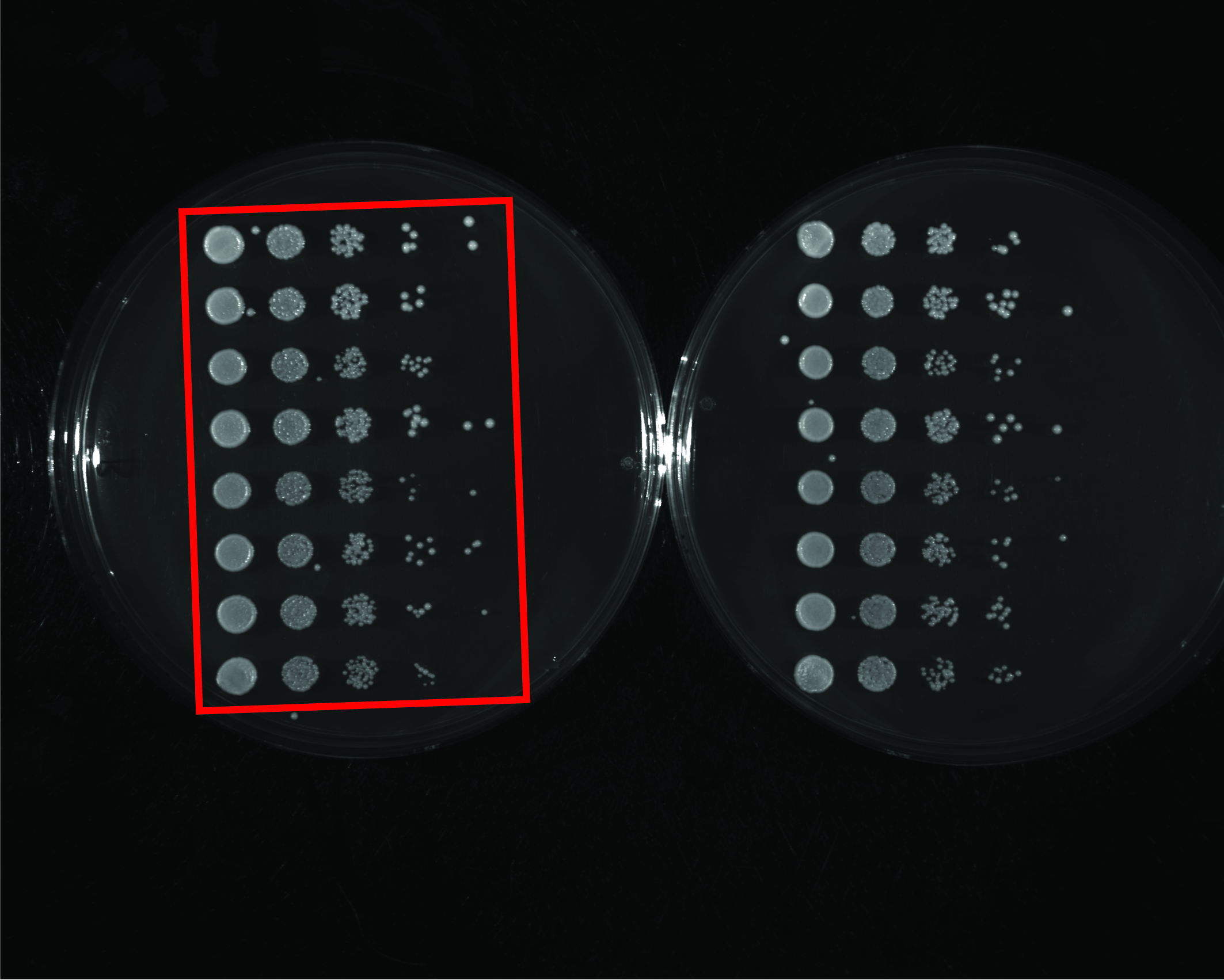

Supplement: Supplementary file 7 — Source data Fig. 3 [file 44319_2025_469_MOESM7_ESM.zip › Figure 3/3D/Passage 2.tif]

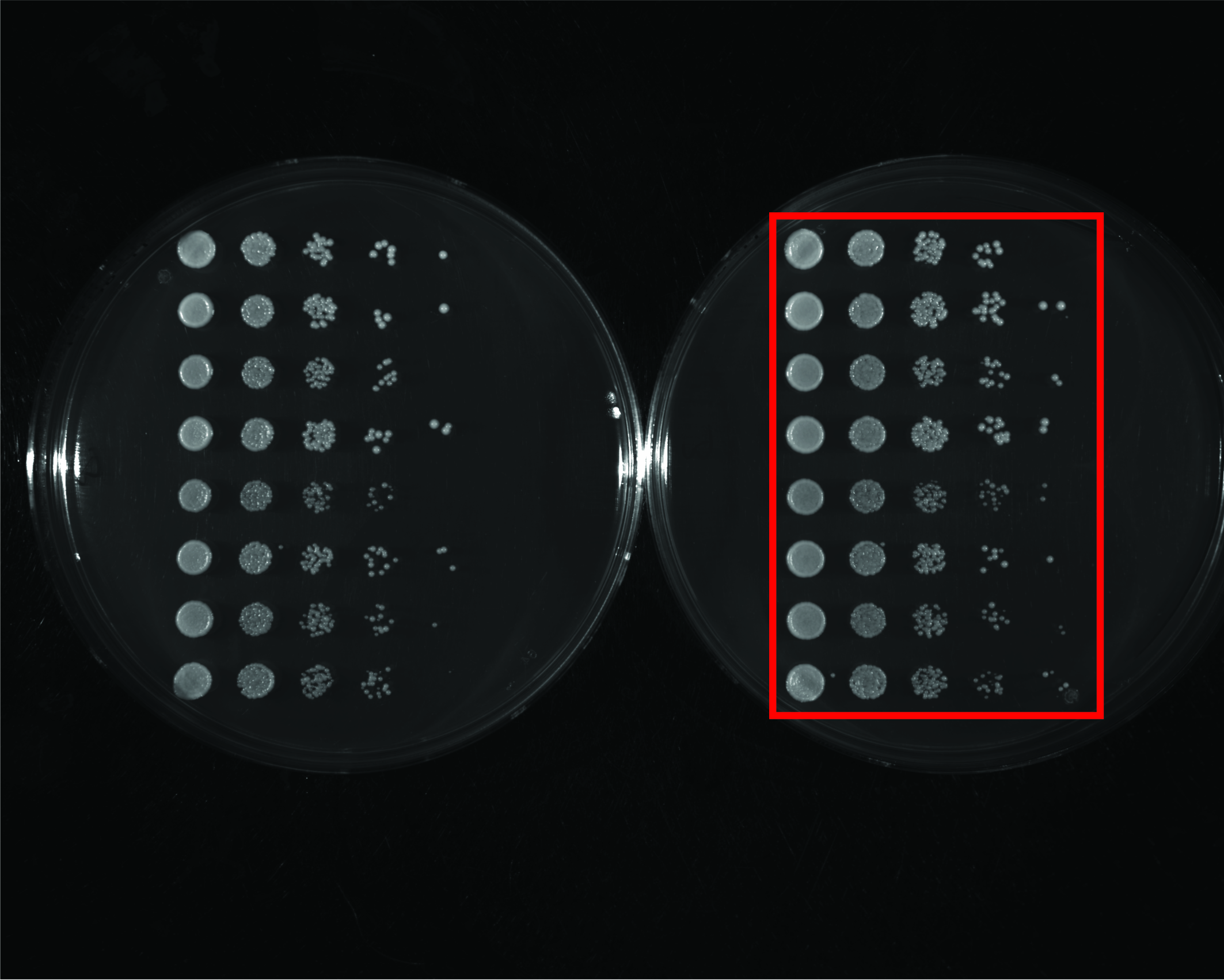

Supplement: Supplementary file 7 — Source data Fig. 3 [file 44319_2025_469_MOESM7_ESM.zip › Figure 3/3D/Passage 3.tif]
