# Supplementary material for: RNase H1 and Sen1 ensure that transient TERRA R-loops promote the repair of short telomeres
Source: EMBO Rep. 2025 May 22;26(12):3032–44. doi: 10.1038/s44319-025-00469-7 (PMC12187912; doi:10.1038/s44319-025-00469-7)
Supplement: Supplementary file 9 — Expanded View Figures [file 44319_2025_469_MOESM9_ESM.pdf]

Expanded View Figures

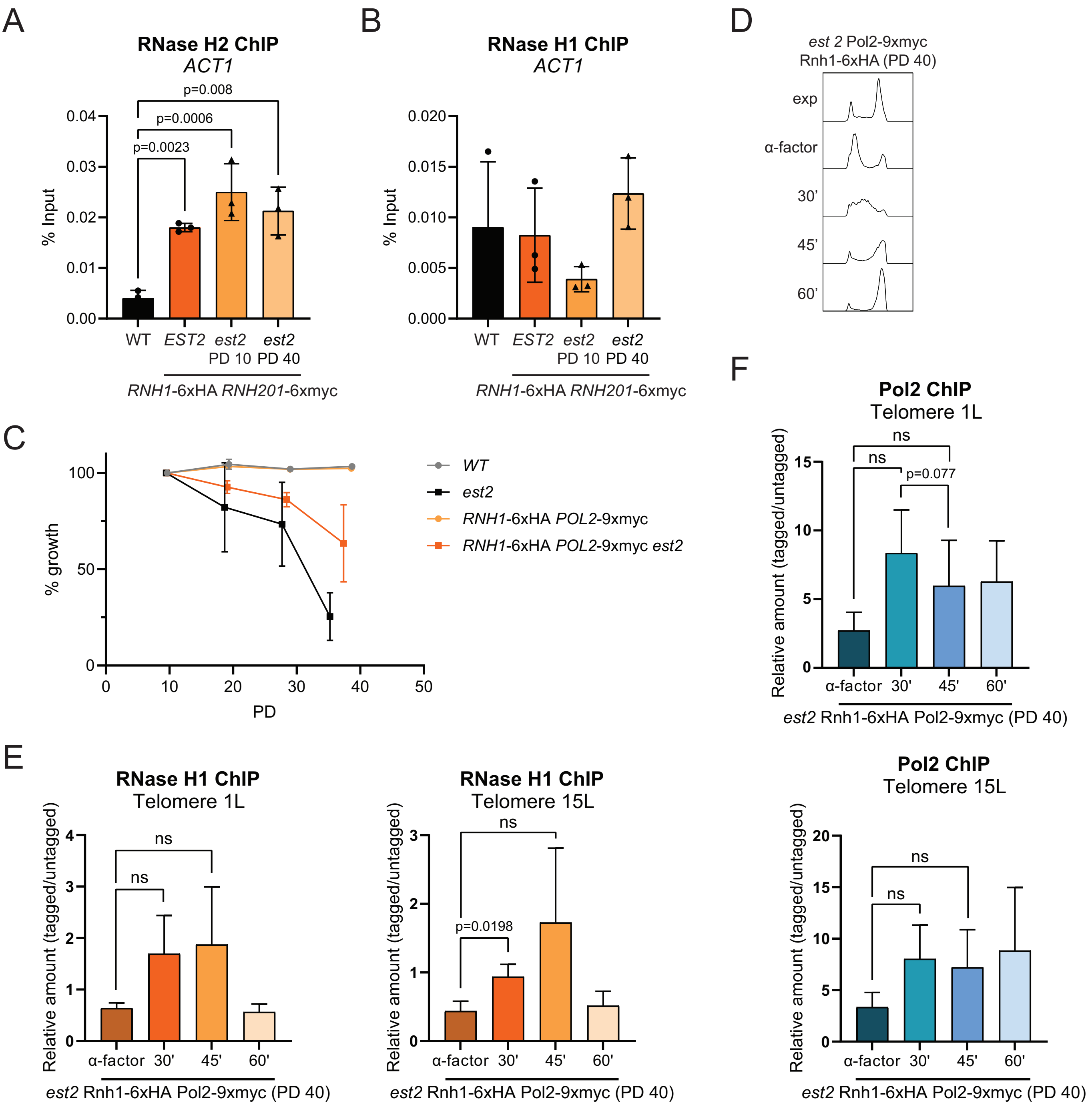

Figure EV1. RNase H1 bind to telomeres in late S/G2 phase of senescent cells.

(**A**) Rnh2 ChIP and (**B**) Rnh1 ChIP performed in exponential cultures of the indicated mutants. Chromatin immunoprecipitation with HA or Myc antibody and qPCR analysis of the indicated strains at Actin locus. Data shown as mean ± SD; $n = 3$. *P*-values were obtained from unpaired t-test. (**C**) Senescence curve was performed in telomerase defective cells (*est2*), and viability was estimated daily by measuring cell culture density, with the first measurement set to 100%. Data shown as mean ± SEM; $n = 3$ biological replicates per genotype. Samples were taken at day 1 (PD 10) and day 4 (PD 40) for DNA content analysis by flow cytometry (**D**) and ChIP (**E**, **F**). Cells were synchronized in G1 phase with α-factor for 2 h and released at 25 °C. (**E**) Rnh1 ChIP and (**F**) Pol2 ChIP performed at the indicated time points of the indicated mutants at telomeres 1 L and 15 L. Chromatin immunoprecipitation with HA or Myc antibody and qPCR analysis of the indicated strains at telomere 1 L and 15 L. Values are represented % input of DNA recovered and relative to untagged strains at correspondent time points. Data shown as mean ± SD; $n = 3$. *P*-values were obtained from two-way ANOVA.

 © The Author(s)

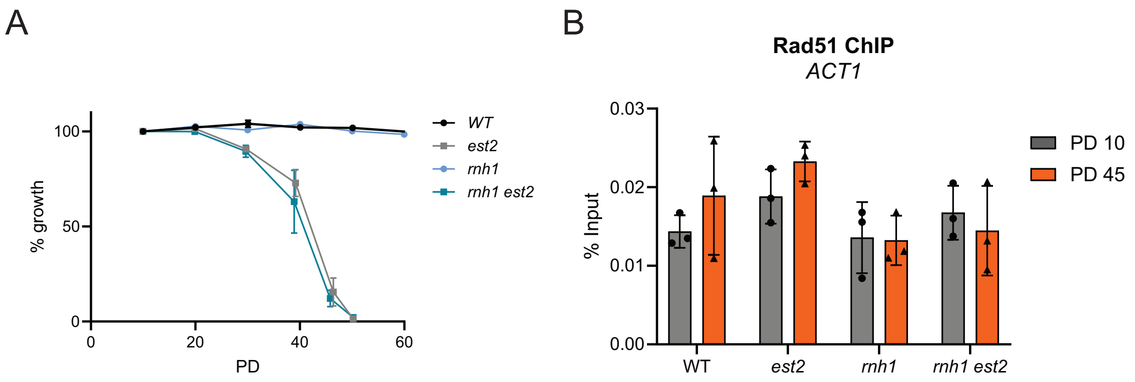

Figure EV2. Rad51 does not bind to Actin locus.

(**A**) Senescence curve was performed in telomerase defective cells (*est2*), and viability was estimated daily by measuring cell culture density, with the first measurement set to 100%. Data shown as mean ± SEM; $n = 3$ biological replicates per genotype. Samples were taken at day 1 (PD 10) and day 5 (PD 45) ChIP (**B**). (**B**) Rad51 ChIP performed at the indicated time points of the indicated mutants. Chromatin immunoprecipitation with HA or Myc antibody and qPCR analysis of the indicated strains at telomere 1 L and 15 L. Values are represented % input of DNA recovered. Data shown as mean ± SD; $n = 3$. *P*-values were obtained from two-way ANOVA.
